# Supplementary material for: dACC response to presentation of negative feedback predicts stimulant dependence diagnosis and stimulant use severity
Source: Neuroimage Clin. 2018 May 9;20:16–23. doi: 10.1016/j.nicl.2018.05.007 (PMC6034587; doi:10.1016/j.nicl.2018.05.007)
Supplement: Supplementary file 1 — Supplementary material [file mmc1.docx]

**Supplement**

**Supplemental Results Page 2**

**Supplemental Table Page 5**

**Table S1**. Brain regions showing significant effects for the contrasts of interest across both SDI and non-SDI.

**Supplemental Figures Page 6**

**Figure S1**. Behavioral performance by group and trial type. a) Deviation from 1000 milliseconds (ms) in participants’ estimates of 1 sec. b) Change in estimate from trial n to n+1 by trial type (n). c) Mean confidence ratings by trial type.

**Figure S2**. Contrasts of interest across non-SDI and SDI. a,c) Informative incorrect > Uninformative incorrect feedback b,d) Informative correct > Informative incorrect. All maps have been cluster corrected using a voxelwise threshold of p < .01, and a cluster level p < .05.

**Supplemental Results**

*Time Estimation Task - Behavioral Results*

The mean (s.d.) proportion of correct feedback for informative feedback trials was 50.5% (5.7%) and for uninformative trials was 51.2% (6.4%). These proportions of feedback suggest that the task worked as intended; performance did not differ on informative and uninformative trials thus allowing us to examine responses to veridical feedback in the absence of expectation differences. Mean absolute deviations (overall mean = 288 msec) from 1-second (presented in Table 2 in the main text) were calculated separately for each group and condition, and were analyzed using a 2 (Group: Cocaine, Control) x 2 (Feedback type: Informative vs. Uninformative) x 2 (Performance: Correct vs. Incorrect) repeated measures ANOVA. This analysis revealed a significant main effect of performance type, which indicated that the mean deviation for correct trials was significantly smaller than that for incorrect trials *F*(1, 69) = 479.68, *p* < .001 (see Supplemental Figure 1a). No significant group effects emerged, suggesting equivalent performance across both groups. Moreover, no two- or three-way interactions reached significance.

Mean ratings of confidence (overall mean = 1.79) were also compared in the context of a 2 (Group: Cocaine, Control) x 2 (Feedback type: Informative vs. Uninformative) x 2 (Performance: Correct vs. Incorrect) repeated measures ANOVA (see Table 2). This analysis revealed a main effect of Performance, such that participants provided higher confidence ratings following accurate compared to inaccurate estimates, *F*(1, 69) = 10.92, *p* = .002. In addition, a Group by Performance interaction reached significance, such that the SDI group had higher confidence after incorrect trials compared to correct trials, a difference that was not present in the non-SDI group *F*(1, 69) = 4.81, *p* = .03. No main effect of group was identified, nor did any other two- or three-way interactions reach significance.

Finally, we examined the magnitude of change in participants’ time estimations (overall mean = 191 msec) following each feedback type by entering their estimation change scores into a 2 (Group: Cocaine, Control) x 2 (Feedback type: Informative vs. Uninformative) x 2 (Performance: Correct vs. Incorrect) repeated measures ANOVA (see Table 2). Main effects of both Group and Feedback were non-significant; however, a main effect of Performance, *F*(1, 69) = 62.94, *p* < .0001 (see Supplemental Figure 1b) indicated that estimates following correct time estimates were changed to a lesser extent than on incorrect time estimation trials. This effect was moderated by a Performance x Feedback interaction, *F*(1, 69) = 3.85, *p* = .05, which indicated that the difference between change after incorrect compared to correct performance was greater when explicit feedback was presented than when ambiguous feedback was presented. No other two-way or three-way interactions reached significance. Of note, no group differences emerged in any of the four conditions (all p's > .2).

*Time Estimation Task – Neuroimaging Contrasts*

As can be seen in Figure S2, *Correct_INFORMED_* feedback resulted in greater response than *Incorrect_INFORMED_* in regions including dACC, bilateral insula, bilateral ventral striatum, and pregenual ACC (see Table 3 in the main manuscript for complete list). Similarly, *Incorrect_INFORMED_* feedback elicited greater response than *Incorrect_UNINFORMED_* feedback in regions including dACC, bilateral insula and pregenual ACC (see Table 3 in the main manuscript for complete list).

*Neuroimaging Results – Effect of Demographic/Abuse Covariates*

As described in the main manuscript, we added age, BDI score, and STAI scores as covariates in relevant regression models, to ensure that group differences in these variables were not responsible for observed effects. As can be seen below, the addition of covariates affected the overall group difference, but the direction of the difference remained in all cases:

Non-SDI > SDI dACC cluster size

BDI (N = 62) 0 voxels at p < .05, 33 & 29 at p<.15

STAI (N = 65) 343 voxels

Age (N = 57) 0 voxels at p < .05, 10 at p<.10

We also ran additional models that included binary (yes/no) variables indicating diagnosis of six other substance dependencies (alcohol, sedative, cannabis, opioid, polydrug, other). As can be seen in the table below, results remained significant in each covariate model, and the addition of these binary variables, alone or in combination did not render the group differences insignificant:

Non-SDI > SDI dACC cluster size

Alcohol: 6 voxels

Sedative: 74 & 52 voxels

Cannabis: 237 voxels

Opioid: 962 voxels

Polydrug: 414 voxels

Other: 6 voxels

All six substances in one model 13 & 3 voxels

In addition to inclusion of covariates in our models, we also attempted to match the groups by selecting a subsample of the SDI group that matched the non-SDI group on both BDI score and gender; the matching process required that only male participants were selected. Running a matched sample of 21 SDI and 21 non-SDI revealed differences at a corrected p of .10 in the *Incorrect_INFORMED_ > Incorrect_UNINFORMED_* contrast, with clusters of 27 and 12 voxels. Because of the difference in proportion of individuals meeting criteria for alcohol dependence, we selected a matched subset of individuals with/without alcohol dependence, and found that at P < .15, there was a cluster of 348 voxels that differed between non-SDI and SDI. Thus, the reported group results appear mostly robust in the face of potential covariates, and appear to highlight differences between the SDI and non-SDI groups that may be specifically attributable to stimulant-related diagnosis/use patterns. Future work that is able to more effectively match on depression and alcohol would be beneficial to understand whether this negative feedback processing difference is due primarily to stimulant use.

*Movement Characteristics*

Below are the relative and absolute motion characteristics for both groups, along with significance tests for group differences in motion. As can be seen, motion was within acceptable limits, and did not differ between groups.

Mean relative motion (SDI): .222 mm

Mean relative motion (non-SDI): .227 mm

t-test of differences: t(69) = .11, p=.91

Mean absolute motion (SDI): .51 mm

Mean absolute motion (non-SDI): .97 mm

t-test of differences: t(69) = .92, p=.36

Table S1. Main Effects and Interactions in the 2 (Performance) x 2 (Feedback) x 2 (Group) analysis.

| Contrast | Region | *k* | x | y | z |
| --- | --- | --- | --- | --- | --- |
| ***Main Effect of Accuracy*** |  |  |  |  |  |
| *Correct > Incorrect* | L occipital pole | 155 | -30 | -92 | -8 |
|  | R occipital pole | 117 | 30 | -94 | -2 |
| *Incorrect > Correct* | none |  |  |  |  |
| ***Main Effect of Information*** |  |  |  |  |  |
| Informative > Uninformative | pgACC/dACC/SMA | 3110 | 10 | 36 | 8 |
|  | L insula/middle frontal gyrus | 2782 | -30 | 14 | -14 |
|  | L inferior parietal lobe | 1115 | -42 | -56 | 36 |
|  | R insula/inferior frontal gyrus | 937 | 28 | 20 | -18 |
|  | Bilateral nucleus accumbens/ putamen/thalamus | 770 | -10 | 8 | -6 |
|  | R middle frontal gyrus | 339 | 44 | 40 | 18 |
|  | R inferior parietal lobe | 329 | 54 | -46 | 44 |
|  | L orbitofrontal cortex | 232 | -42 | 54 | -4 |
|  | R precentral gyrus | 5 | 52 | 10 | 26 |
| Uninformative > Informative | none |  |  |  |  |
| ***Main effect of Group*** |  |  |  |  |  |
| SDI > Non-SDI | none |  |  |  |  |
| Non-SDI > SDI | none |  |  |  |  |
| ***Accuracy x Information Interaction*** | |  |  |  |  |
|  | L occipital pole | 155 | 30 | -90 | -4 |
|  | R occiptal pole | 118 | -28 | -92 | -4 |

**Figure S1**. Behavioral performance by group and trial type. a) Deviation from 1000 milliseconds (ms) in participants’ estimates of 1 sec. b) Change in estimate from trial n to n+1 by trial type (n). c) Confidence ratings by trial type.

|   a |
| --- |
|   b |
|  |

**Figure S2**. One-sample contrasts of interest within non-SDI and SDI groups, respectively. Panels a and c display each Incorrect_INFORMED_ > Incorrect_UNINFORMED_ contrasts, while panels b and d display each Incorrect_INFORMED_ > Correct_INFORMED_ contrast. Hot colors indicate regions showing greater activity than baseline; cold colors indicate regions showing reduced activity compared to baseline. Maps are displayed using a corrected threshold of p < .05 using threshold free cluster enhancement with 5000 permutations.

| 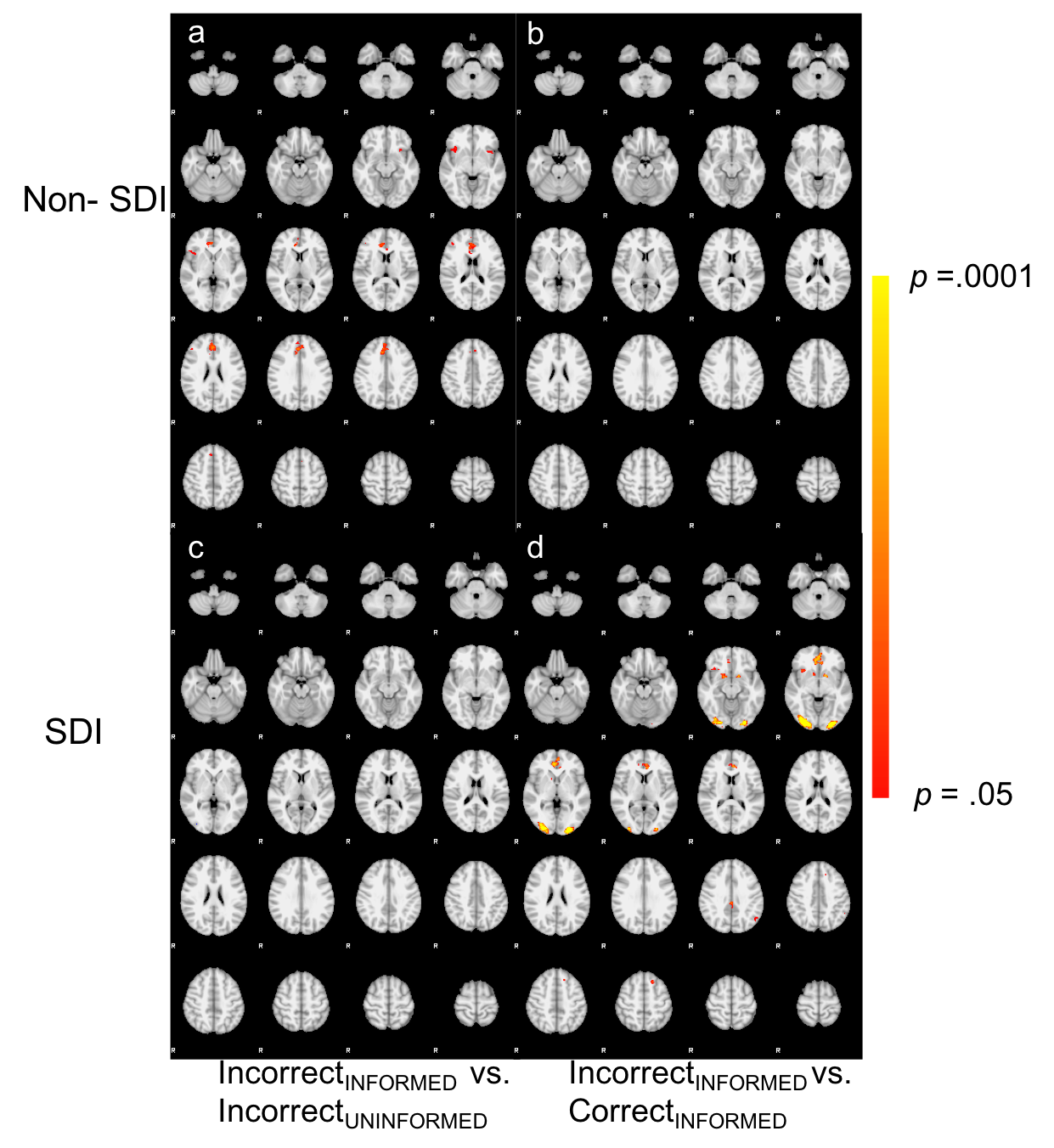 |
| --- |
